# Forest type effects on the retention of radiocesium in organic layers of forest ecosystems affected by the Fukushima nuclear accident

**DOI:** 10.1038/srep38591

**Published:** 2016-12-15

**Authors:** Jun Koarashi, Mariko Atarashi-Andoh, Takeshi Matsunaga, Yukihisa Sanada

**Affiliations:** 1 Nuclear Science and Engineering Center, Japan Atomic Energy Agency, Ibaraki 319-1195, Japan; 2 Fukushima Environmental Safety Center, Japan Atomic Energy Agency, Fukushima 975-0036, Japan

## Abstract

The Fukushima Daiichi nuclear power plant disaster caused serious radiocesium (^137^Cs) contamination of forest ecosystems over a wide area. Forest-floor organic layers play a key role in controlling the overall bioavailability of ^137^Cs in forest ecosystems; however, there is still an insufficient understanding of how forest types influence the retention capability of ^137^Cs in organic layers in Japanese forest ecosystems. Here we conducted plot-scale investigations on the retention of ^137^Cs in organic layers at two contrasting forest sites in Fukushima. In a deciduous broad-leaved forest, approximately 80% of the deposited ^137^Cs migrated to mineral soil located below the organic layers within two years after the accident, with an ecological half-life of approximately one year. Conversely, in an evergreen coniferous forest, more than half of the deposited ^137^Cs remained in the organic layers, with an ecological half-life of 2.1 years. The observed retention behavior can be well explained by the tree phenology and accumulation of ^137^Cs associated with litter materials with different degrees of degradation in the organic layers. Spatial and temporal patterns of gamma-ray dose rates depended on the retention capability. Our results demonstrate that enhanced radiation risks last longer in evergreen coniferous forests than in deciduous broad-leaved forests.

As a result of the accident at the Fukushima Daiichi nuclear power plant (NPP) in March 2011, more than several tens of thousands of hectares of forests in Japan experienced severe radioactive contamination^1^. Of the radionuclides found in the atmospheric fallout from the accident, radiocesium (^137^Cs), with a half-life of 30.1 years, is the primary source of concern, because over the coming decades, forest ecosystems contaminated with ^137^Cs will enhance the radiation exposure of the local population via both the elevated ambient air dose rate (external exposure) and the consumption of contaminated forest products (internal exposure)^2^.

One of the most important lessons learned from observational studies of ^137^Cs migration in European forests after the Chernobyl NPP accident was that forest-floor organic layers retain the largest portion of the fallout ^137^Cs for a long time (over a decade) because they are a prolonged source for ^137^Cs recycling (i.e., the uptake of ^137^Cs by trees from the organic layers and the subsequent redeposition of ^137^Cs onto the forest floor via litterfall) in forest ecosystems^3^,^4^,^5^,^6^,^7^. This is primarily because organic layers are deficient in clay minerals that offer specific sites for ^137^Cs adsorption^8^,^9^, and therefore, ^137^Cs in these layers is not strongly fixed and remains potentially mobile and bioavailable^7^,^10^. However, it is well documented that once ^137^Cs is transferred to the mineral soil located below the organic layers, it is rapidly immobilized in the upper layers of the mineral soil via its interactions with clay minerals^11^,^12^. The fixation of ^137^Cs by clay minerals in the mineral soil results in a reduced availability for ^137^Cs uptake by plant roots^4^,^13^,^14^. The striking contrasts in the mobility and bioavailability of ^137^Cs between the organic and mineral soil layers suggests that the retention of ^137^Cs in organic layers is a key factor in evaluating radiation risks delivered from ^137^Cs contamination of forest ecosystems.

Deciduous broad-leaved forests (49.1% of the forested area) and evergreen coniferous forests (47.0%) are the dominant forest types in the area heavily affected by the Fukushima NPP accident in Japan^1^. These forests greatly differ in their tree phenology, which is one of the most important drivers of ecosystem processes, and therefore, it is very likely that the behavior of ^137^Cs in the forest ecosystems after atmospheric fallout differs depending on the forest type. The tree canopies of evergreen coniferous forests acted as efficient filters of the atmospheric plume of ^137^Cs from the Fukushima NPP accident; therefore, a large proportion of ^137^Cs was initially intercepted and retained by the tree canopies and subsequently transferred to the forest floor via processes such as throughfall, stemflow, and litterfall^15^,^16^. By contrast, the canopy-interception effect was less important in deciduous broad-leaved forests compared to evergreen coniferous forests because the deciduous forests were leafless at the time of the accident; therefore, a large proportion of ^137^Cs delivered by the Fukushima NPP accident was directly deposited on the forest floor^16^,^17^,^18^. Therefore, it is easily assumed that the differences in ^137^Cs deposition behavior between the forests will result in different capacities of ^137^Cs retention in their organic layers, at least over the first several years following forest contamination.

The rate of decomposition of litter materials in organic layers and the subsequent release of bioavailable ^137^Cs are also important factors in controlling the rate of ^137^Cs recycling within a forest ecosystem, and these factors are largely dependent on the forest type. Deciduous leaf fall is an annual occurrence and the decomposition of broad leaves in such forests has been shown to be rapid^19^,^20^, whereas the mean leaf longevity of evergreen coniferous trees is longer than a year^21^ and the decomposition of needle-like leaves appears to be much slower compared to broad leaves^22^,^23^. The rate of decomposition of litter materials in organic layers is also influenced by climatological factors such as temperature and precipitation. Therefore, it is suggested that the behavior of ^137^Cs in organic layers differs between European forests (affected by the Chernobyl NPP accident) and Japanese forests (affected by the Fukushima NPP accident)^24^,^25^,^26^; studies under specific climatological and ecological conditions in Japan are urgently required to assess the environmental consequences of the Fukushima NPP accident. In addition, forests have complex stand structures and microtopography, and therefore, the quantity and quality (degrees of degradation) of litter materials accumulated in organic layers are highly spatially variable, even within a forest ecosystem^27^. Spatial heterogeneity can also be a complicating factor in quantitatively assessing the retention capability of organic layers for ^137^Cs and its dependence on the forest type^20^,^28^.

Therefore, even though forest-floor organic layers are of key importance in ^137^Cs cycling in forest ecosystems, there is still an insufficient understanding of how much and for how long organic layers can retain ^137^Cs in Japanese forest ecosystems affected by the Fukushima NPP accident. To explore the role of organic layers in ^137^Cs retention, we conducted plot-scale investigations at two contrasting forest sites in Fukushima, Japan (Fig. 1). We collected samples from the organic and underlying mineral soil (0–5 cm) layers at 25 locations within each of the 20 m × 20 m plot areas established in the deciduous broad-leaved forest (DBF) and evergreen coniferous (Japanese cedar-dominated) forest (CF). Litter samples in organic layers were collected separately from the upper L layer (litter layer, consisting of intact and relatively undecomposed leaves) and the lower F layer (fermentation layer, consisting of partially and well-degraded plant residues). The sample collections were conducted in December 2012 (21 months after the Fukushima NPP accident) at the DBF site and in August 2013 (29 months after the accident) at the CF site, and the collected samples were analyzed for radiocesium isotopes (^137^Cs and ^134^Cs) and for organic carbon (C) and total nitrogen (N). Based on the results, we evaluated the forest type effects on, and plot-scale spatial variability in, the retention behavior of ^137^Cs in the organic layers of Japanese forest ecosystems to obtain insights into the radioecological consequences of the Fukushima NPP accident.

## Results

### Radiocesium concentrations

At both forest sites, ^137^Cs concentrations were higher in the organic layer (L and F layers) than in the topsoil (0–5 cm) layer (Table 1; also see Tables S1 and S2 in Supplementary information). Within the organic layer, the concentrations showed a different depth-wise pattern between the sites; ^137^Cs was specifically concentrated in the lower F layer at the DBF site, whereas ^137^Cs concentration was fairly similar between the L and F layers at the CF site.

The measured ^137^Cs and ^134^Cs concentrations showed a similar pattern of distribution for all samples. The ^134^Cs/^137^Cs activity ratios were 0.56 ± 0.03 and 0.46 ± 0.02 (mean ± standard deviation) for the litter and soil samples collected at the DBF and CF sites, respectively (Table 1). The ratios were close to those (0.57 and 0.46, respectively) theoretically predicted for Fukushima-derived radiocesium at the time of sample collection, the initial ratio being unity in March 2011 and decreasing according to different rates of radioactive decay (the physical half-lives of ^137^Cs and ^134^Cs are 30.1 and 2.1 years, respectively). The ratios indicate that the radiocesium isotopes observed in the present study originate from the Fukushima NPP accident. For simplicity, we will discuss only ^137^Cs results in the following parts of this paper.

### Total inventory of ^137^Cs in the forest surface soils (organic and topsoil layers)

The total inventories of ^137^Cs in the forest surface soils (organic and topsoil layers) were 49.8 ± 11.0 kBq m^−2^ (mean ± standard deviation, n = 25) at the DBF site and 43.8 ± 12.1 kBq m^−2^ (n = 25) at the CF site (Table 1), and showed spatially heterogeneous distributions within the 20 m × 20 m plot areas (Figs 2b and 3b). The difference in the total ^137^Cs inventory between the sites was not considered to be statistically significant (P = 0.07 via the unpaired t-test). Coefficients of variation (CV) values of the total ^137^Cs inventory, calculated as the ratio of the standard deviation to the mean (i.e., relative standard deviation), were 22.1% and 27.5% for the DBF and CF sites, respectively. In both plots, the spatial distribution of the total ^137^Cs inventory was not found to be related to that of the standing trees (Figs 2 and 3). There was no statistically significant (at a 5% significance level) correlation between the total ^137^Cs inventory and the stand basal area in the 4 m × 4 m subplot (calculated as the sum of the cross-sectional areas at breast height for all trees in the target subplot) (Table 2).

### Inventory of ^137^Cs in organic (L and F) layers

Although the total ^137^Cs inventories in the surface soils at the two sites were similar, the inventories in the organic (L + F) layers were quite different. The inventories of ^137^Cs in the organic layers were 10.0 ± 3.2 kBq m^−2^ (CV: 31.9%) and 23.0 ± 5.5 kBq m^−2^ (CV: 24.1%) at the DBF and CF sites, respectively (Table 1). At the DBF site, a large proportion (78.8 ± 8.4%) of the total ^137^Cs inventory was found in the topsoil layer below the organic layer. By contrast, at the CF site, more than half (54.0 ± 12.2%) of the total inventory still remained in the organic layer, even though the sample collection at this site was conducted approximately 8 months after the sample collection at the DBF site.

The ^137^Cs inventory in organic layers has been investigated several times at these sites since the accident in March 2011^29^,^30^. This allows us to assess the temporal changes in the ^137^Cs inventory in the layers during the first three-year period (Fig. 4). The ^137^Cs inventory in the organic layers decreased with time at both sites and this decreasing pattern can be characterized by an exponential decay model:


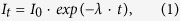


where *I*_*t*_ is the ^137^Cs inventory (kBq m^−2^) in the organic layer at time *t, I*_*0*_ is the ^137^Cs inventory (kBq m^−2^) in the layer at time *t* = 0, *λ* is the decay constant (y^−1^), and *t* is the elapsed time (y) since the accident. The effective half-life (*T*_*eff*_ in years) of ^137^Cs in the organic layer can, therefore, be calculated as *T*_*eff*_ = ln(2)/*λ*. Because the ^137^Cs inventory data presented in Fig. 4 are decay-corrected to the sampling date, the effective half-life is a measure of the combined effect of physical (radioactive) decay and ecological elimination processes. The ecological half-life (*T*_*e*_ in years) of ^137^Cs in the organic layer can, therefore, be evaluated as


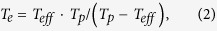


where *T*_*p*_ is the physical half-life of ^137^Cs (30.1 years). The ecological half-lives of ^137^Cs estimated in this way were 0.95 years (r = 0.90) and 2.1 years (r = 0.98) for the organic layers at the DBF and CF sites, respectively.

The spatial distribution of the ^137^Cs inventory in the organic layer showed a similar pattern to that of the total ^137^Cs inventory at the CF site (Fig. 3), and there was a significant positive (r = 0.49, p < 0.05) correlation between the two inventories (Table 2). Contrastingly, the total and organic-layer ^137^Cs inventories showed different spatial distribution patterns at the DBF site (Fig. 2), and no correlation was found between the two. Similar to the total ^137^Cs inventory, the ^137^Cs inventory in the organic layer showed no correlation with the stand basal area in the target subplots at both sites (Table 2). The inventory of ^137^Cs in the organic layer was positively (r = 0.88, p < 0.0001) correlated with that of litter materials in the layer across the two sites (Fig. 5a).

Within the organic layer, the lower F layer accumulated more ^137^Cs than the upper L layer at the DBF site, whereas the L layer accumulated more ^137^Cs at the CF site (Table 1). For each layer, there was a significant (p < 0.01) positive correlation between the ^137^Cs inventory and the litter-material inventory in the layer (Fig. 5b; r = 0.74 and r = 0.84 for the L and F layers of the DBF, respectively, and r = 0.65 and r = 0.61 for the L and F layers of the CF, respectively). At the CF site, the ^137^Cs inventory appeared to increase linearly with increasing litter-material inventory regardless of the layer (L or F). At the DBF site, however, the ^137^Cs inventories for the L and F layers were not observed to be approximated by a single linear relationship. These observations indicate that the larger accumulation of ^137^Cs in the organic layer at the CF site can be explained simply by the larger accumulation of litter materials on the forest floor, while the larger accumulation of ^137^Cs in the organic layer at the DBF site depends more on the larger accumulation of highly ^137^Cs-contaminated materials in the F layer.

### Carbon and nitrogen contents

At both sites, the L layer, consisting of intact and relatively undecomposed leaves, showed a high C content (>440 gC kg^−1^ dw, Table 1), indicating that the litter materials in the L layers have originated primarily from recent litterfall events. The C content in the topsoil was much lower than that in the organic layer, which was likely due to the mixing of mineral soil particles with organic materials in the topsoil layer. The carbon-to-nitrogen (C/N) ratio generally decreased with depth, indicating that the litter materials in the F layers were older and had experienced higher degrees of microbial decomposition compared to those in the L layers^31^,^32^. This was consistent with the visual aspect of the litter materials collected from the F layers as a mixture of finely fragmented plant residues and macroscopically unrecognizable organic materials^20^.

### Gamma-ray dose rates at the ground surface

Gamma-ray dose rates were measured at the ground surface at both sites using a plastic scintillation fiber (PSF) detection system, and the spatial distributions of the dose rate in the 20 m × 20 m plot areas were recorded (Figs 2 and 3). At the DBF site, the gamma-ray dose rates tended to be higher in areas with a larger accumulation of ^137^Cs in the surface soil (organic and topsoil layers). At the CF site, however, the dose rates seemed to be more related to ^137^Cs accumulation in the organic layer, rather than in the surface soil. The gamma-ray dose rates were significantly (p < 0.01 via the unpaired t-test) higher at the CF site (0.34 ± 0.04 μSv h^−1^) than at the DBF site (0.26 ± 0.09 μSv h^−1^).

## Discussion

Organic layers on the forest floor are a dynamic component of forest ecosystems and play a key role in ecosystem functioning via the retention and cycling of nutrients^33^. Our plot-scale investigations conducted at two contrasting forests in Fukushima show that organic layers also play a significant role in the retention, and therefore, the behavior of ^137^Cs in forest ecosystems. In the DBF, approximately 80% of the ^137^Cs that had been deposited onto the forest floor migrated to the underlying mineral soil within two years of the accident (Table 1). Contrastingly, in the CF, more than half of the deposited ^137^Cs was retained in the organic layer even two and a half years after the accident. The ecological half-life of ^137^Cs in the organic layers was estimated to be 2.1 years at the CF site, which was approximately twice as long as that at the DBF site (Fig. 4). Overall, the correlations found between ^137^Cs and the litter-material inventories in the organic layers across the two sites (Fig. 5a) indicate that, in general, the denser (or thicker) organic layers of the CF more efficiently retain ^137^Cs on the forest floor than the less-dense (or thinner) organic layers of the DBF^4^,^28^,^29^,^34^.

The separate sampling and analysis of the L and F layers provided more detailed information concerning the accumulation of litter-associated ^137^Cs within the organic layers and revealed that the retention processes of ^137^Cs in the organic layers differed markedly between the two forest sites. The retention processes of ^137^Cs in organic layers appeared tightly linked to the ecosystem processes in the two contrasting forests. In the organic layers of the DBF, ^137^Cs was primarily retained in association with highly degraded (evidenced by the C/N data) litter materials in the lower F layers (Table 1 and Fig. 5b). In temperate DBFs in Japan, the bulk of the litterfall occurs in autumn (October and November) and new leaves begin to expand in spring (April and May). Trees at the DBF site had no leaves in March 2011 when the Fukushima NPP accident occurred, and therefore, it is assumed that most of the Fukushima-derived ^137^Cs was directly deposited onto forest-floor litter materials^17^,^18^. Because broadleaf litter is known to be relatively rapidly decomposed via microbial activity in the organic layers (a mean residence time of a few years)^19^,^20^,^31^, it is likely that the litter materials contaminated by the direct deposition of ^137^Cs have been degraded since March 2011 and that most of the ^137^Cs was leached from the organic layers and then immobilized in the topsoil, with a minor fraction (14% of the total deposition) being still retained in the F layers in December 2012^17^,^18^,^35^.

At the time of sample collection in the DBF, the annual litterfall in 2012 was complete and the litter materials (intact and relatively undecomposed leaves) in the L layer were primarily from the most recent litterfall events. The ^137^Cs concentration of the litter materials in the L layers was significantly lower than that in the F layers (Table 1), but three orders of magnitude higher than the pre-accident level, indicating that the newly emerged leaves in 2012 were contaminated with ^137^Cs via mechanisms such as translocation from tree stems and uptake by roots from the soil^36^,^37^. However, the short ecological half-life (0.95 years) of ^137^Cs in the organic layers at this site suggests a rapid decrease in the inventory of potentially mobile ^137^Cs in the organic layers, and therefore, a rapid reduction in ^137^Cs recycling in the soil–plant system^4^,^14^. This scenario for the DBF site is consistent with the rapid decrease in ^137^Cs concentration in fresh broad leaves, which has been observed at various locations in Japan following the Fukushima NPP accident^20^,^37^,^38^. It has also been observed that in a DBF in Japan, the amount of ^137^Cs that migrated downward through the litter–mineral soil boundary decreased with time at a rate of approximately 50% per year during the period of April 2012–May 2015^25^. This reduction rate for ^137^Cs migration is in good agreement with the estimated ecological half-life of ^137^Cs in the organic layers at our DBF site.

In contrast to the DBF, the organic layers of the CF showed a high retention capability for ^137^Cs, which was primarily dependent on the accumulation of less-degraded, highly contaminated litter materials in the L layers (Table 1 and Fig. 5b). This high retention capability is related to the tree phenology at this site and can be reasonably explained by considering that leaves on the cedar trees, as well as the litter materials on the forest floor, were directly contaminated with ^137^Cs as a result of fallout from the Fukushima NPP accident in March 2011. Since then, a portion of the contaminated leaves has gradually been transferred to the forest floor via litterfall over time, and most of the ^137^Cs of litterfall origin remained in the L layers until August 2013. This migration pattern is considered reasonable given the observation that more than 60% of the Fukushima-derived ^137^Cs was initially intercepted by the tree canopy in evergreen coniferous forests^15^. This pattern is also consistent with the leaf longevity of Japanese cedars (4–8 years)^21^,^39^. The lagged (indirect) input of the ^137^Cs-contaminated leaves to the forest floor^16^, in combination with a possible slower microbial decomposition of needle-like cedar leaves in the organic layers compared to broad leaves^22^,^23^, is the most likely explanation for the observed higher retention (or longer ecological half-life) of ^137^Cs in the organic layers at the CF site compared to that at the DBF site. The results also suggest that additional inputs of leaf-associated ^137^Cs to the forest floor over the next several years are possible.

The effective retention of ^137^Cs (as potentially mobile ^137^Cs) in the thick organic layers of the CF may facilitate the recycling of ^137^Cs via ^137^Cs uptake by trees from the organic layers and its subsequent re-deposition onto the forest floor via litterfall^5^,^40^. The scenario for the CF site highly contrasts that for the DBF site (see above), demonstrating that the forest type significantly influences both the short- and long-term behaviors of ^137^Cs within a forest ecosystem^7^,^39^, and clearly, the retention of ^137^Cs in organic layers plays a key role in controlling the overall ^137^Cs behavior^5^,^26^. The observations conducted in European forests after the Chernobyl NPP accident have consistently shown that a large proportion of ^137^Cs persisted in forest organic layers for over a decade, resulting in long-lasting ^137^Cs bioavailability in the soil–plant system^3^,^4^,^5^,^6^,^7^,^28^. This may be partly due to climatological factors such as temperature and precipitation, both of which can affect the microbial decomposition of litter materials in organic layers^23^,^24^,^41^. For example, a mean residence time of 28–42 years for litter materials has been reportedly observed for organic layers in a spruce-dominated forest in Scandinavia^42^, which is noticeably longer than that of several years observed for the organic layers of DBFs in Japan^19^.

The ^137^Cs inventory in the organic layers was spatially variable at the plot scale (20 m × 20 m) in both forests, with CV values of 32% and 24% for the DBF and CF sites, respectively. In the present study, the spatial distribution of the ^137^Cs inventory in the organic layers did not show any clear relationship to that of the standing trees and stand basal area (Figs 2 and 3 and Table 2), suggesting that the forest stand structure was not a factor causing the spatial heterogeneity of the ^137^Cs inventory in the organic layers. Instead, the correlations observed between the ^137^Cs and litter-material inventories (Table 2 and Fig. 5) suggest that the accumulation and reduction processes of litter materials on the forest floor were more significant in creating the spatial variability in the ^137^Cs inventory in the organic layers^20^. The accumulation and reduction processes include the lateral transport of leaves via wind action while falling and the redistribution of fallen leaf litter on the forest floor^27^,^43^,^44^. The decomposition rate of litter materials on the forest floor, which can be modified by microclimate-related microbial activities and soil invertebrate activities, is also a possible factor influencing the increase and decrease in the amount of litter materials in the organic layers^45^,^46^.

The retention properties of the organic layers for ^137^Cs also exerted a significant influence on the spatial and temporal patterns of the gamma-ray dose rate at the ground surface. In the CF where a large proportion of the deposited ^137^Cs had been retained in the organic layers, the spatial pattern of the gamma-ray dose rate was found to be relevant to that of the ^137^Cs inventory in the organic layers (Fig. 3). In the DBF where approximately half of the deposited ^137^Cs had already migrated to the mineral soil, the spatial pattern of the gamma-ray dose rate was found to be more relevant to that of the total ^137^Cs inventory, rather than the organic-layer ^137^Cs inventory (Fig. 2). The gamma-ray dose rate was significantly higher at the CF site than at the DBF site, even though the total inventory of ^137^Cs in the surface soils (organic and topsoil layers) was similar for both sites (Table 1). This is likely a result of the shielding effect because gamma radiation from ^137^Cs in deeper soil layers is more efficiently attenuated by the overlying soil^47^.

In conclusion, our study demonstrates that the forest type strongly affects the retention capability of organic layers for ^137^Cs in conjunction with the litter dynamics, and subsequently, affects the mobility and bioavailability of ^137^Cs within a forest ecosystem. The forest-type-dependent retention capability indicates that enhanced radiation risks delivered from both internal (via consumption of forest products) and external exposures to the local population last longer in CFs than in DBFs. The spatial and temporal patterns of the gamma-ray dose rate and their control factor also depend on the forest type. Continuous observations of the ^137^Cs inventory in the organic layers in these two contrasting sites will help improve our understanding of the retention behavior of ^137^Cs in organic layers of forest ecosystems in Japan, which is the key to accurately assess both short- and long-term radioecological impacts of the Fukushima NPP accident.

## Methods

### Study sites

**Figure 1:**
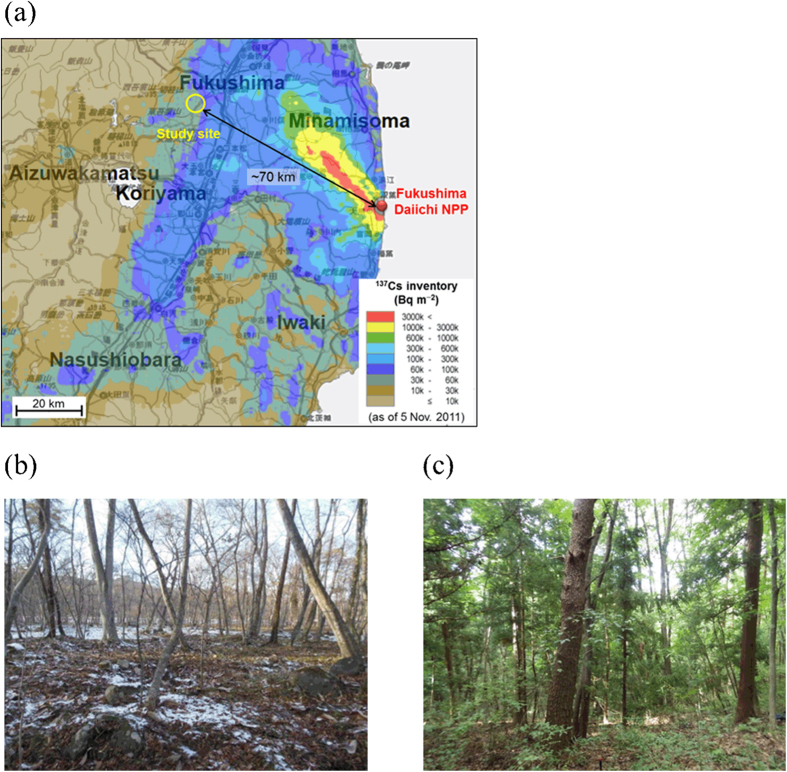
(**a**) Location of the study site and photographs of (**b**) the DBF and (**c**) CF investigated in this study. The ^137^Cs inventory map was generated using the website “Extension Site of Distribution Map of Radiation Dose, etc.” prepared by MEXT, Japan^53^. Photographs were taken at the time of sample collection by T. Matsunaga.

This study was conducted at two forest sites (Fig. 1) located in the southwestern part of the city of Fukushima (37.71°N, 140.36°E), approximately 70 km northwest of the Fukushima Daiichi NPP. The first site was a DBF dominated by Japanese oak and hornbeam, located along a riverbank. The second site was a CF dominated by Japanese cedar, located along a trail over a small mountain. The distance between the two sites is approximately 1.5 km. The soils have been classified as Fluvisols and Andosols for the DBF and CF sites, respectively, using the classification of the Food and Agriculture Organization^48^. The mean annual temperature and precipitation for the last 10 years at the Fukushima meteorological station are 13.3 °C and 1234 mm, respectively (Japan Meteorological Agency).

The vertical distribution and retention processes of the Fukushima-derived radiocesium in the organic and mineral soil layers of these sites have been studied since the accident in March 2011^29^,^30^,^49^. The DBF and CF sites in this study correspond to the sites represented as FR-2 and FR-5, respectively, in the previous studies. For more detail on the site characteristics, including the physicochemical properties of the soils, see Koarashi *et al*.^29^.

### Sample collection and treatment

**Figure 2:**
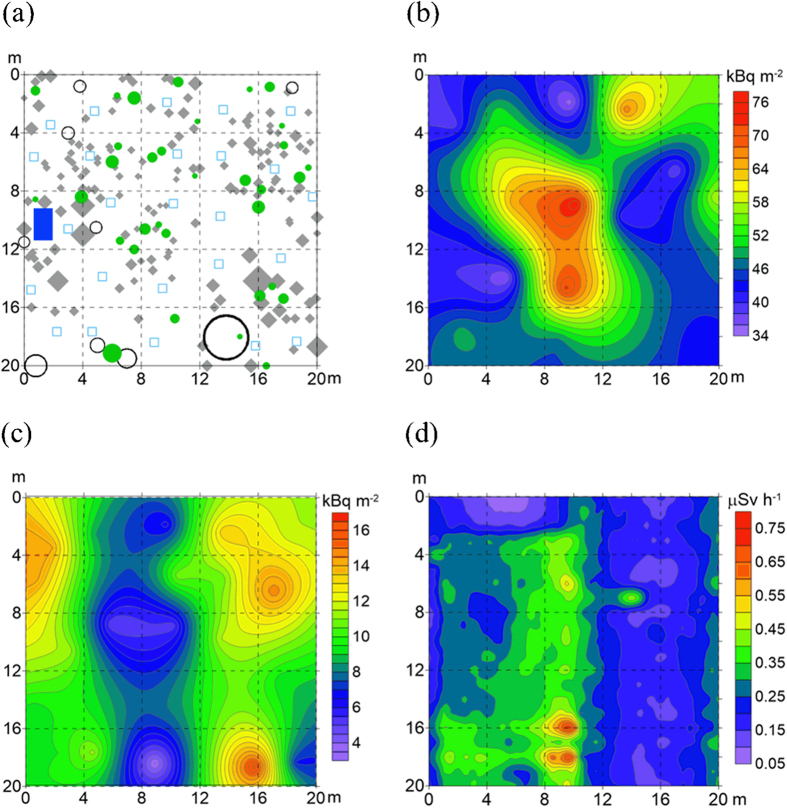
Spatial distribution maps of (**a**) the sampling points and standing trees, (**b**) the total ^137^Cs inventory in the organic and topsoil layers, (**c**) the ^137^Cs inventory in the organic layer, and (**d**) the gamma-ray dose rate at the ground surface in the 20 m × 20 m plot area of the DBF. In (**a**), open squares indicate the sampling points, filled circles indicate the standing trees (represented by their breast height diameters), filled diamonds indicate the stones (larger than 10 cm in diameter), open circles indicate the hollows in the land, and the filled rectangle indicates the area covered by a vinyl sheet.

**Figure 3:**
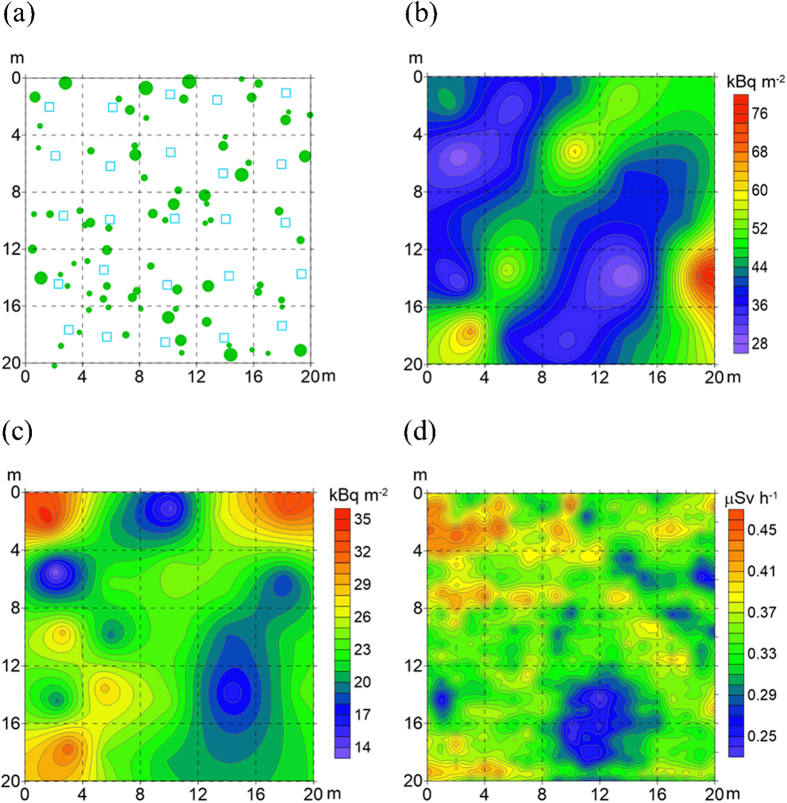
Spatial distribution maps of (**a**) the sampling points and standing trees, (**b**) the total ^137^Cs inventory in the organic and topsoil layers, (**c**) the ^137^Cs inventory in the organic layer, and (**d**) the gamma-ray dose rate at the ground surface in the 20 m × 20 m plot area of the CF. In (**a**), open squares indicate the sampling points and filled circles indicate the standing trees (represented by their breast height diameters).

**Figure 4:**
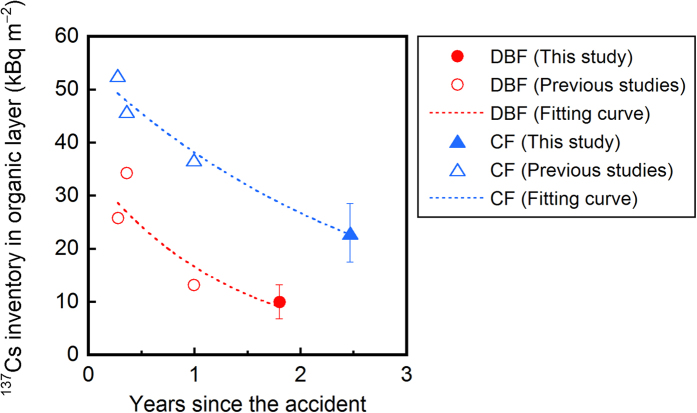
Temporal changes in the ^137^Cs inventory in the organic (L + F) layers of the two contrasting forests since the Fukushima NPP accident. The data are decay-corrected to the sampling date. Previous studies were conducted in June 2011^29^, July 2011^30^, and March 2012 (Koarashi *et al*., unpublished).

**Figure 5:**
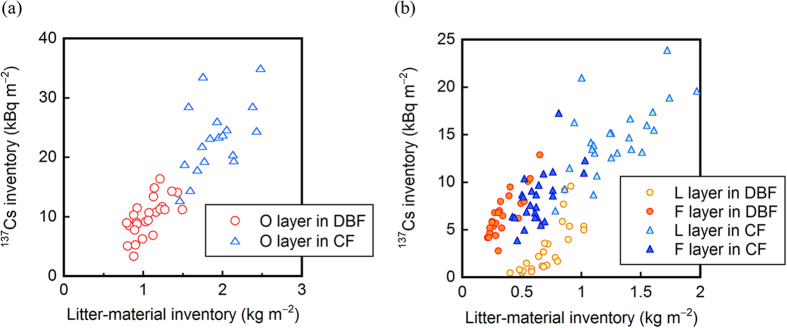
Correlations between the ^137^Cs and litter-material inventories in (**a**) the organic (L + F) layer and (**b**) the separated L and F layers at the DBF and CF sites.

Field samples were collected in December 2012 (21 months after the accident) at the DBF site and in August 2013 (29 months after the accident) at the CF site. At the time of sample collection in the DBF, the trees had no leaves due to autumn leaf fall (litterfall) in 2012 and the forest floor was slightly covered with snow (see Fig. 1(b)). A 400 m^2^ (20 m × 20 m) plot was established at each site and was divided into 25 subplots each having an area of 4 m × 4 m. Litter samples in the organic layer (the upper L and the lower F layers, separately) were collected manually from an area of 900 cm^2^ randomly selected within each of the 25 subplots (but generally located near the center of each of the 25 subplots; see Figs 2a and 3a). Three replicate topsoil (0–5 cm) samples were then collected using a cylindrical soil sampler (5 cm in diameter and 5 cm in depth) from the soil surface where the organic layer was removed. The depth range (0–5 cm) of the soil sampling was chosen on the basis of our previous studies, which showed that most (>94%) of the total ^137^Cs inventory was located within the organic and the upper 5-cm soil layers at the sites^29^,^30^. At each site, the sampling locations and forest stand descriptions (locations and breast height diameters of the standing trees) were recorded. The DBF site was stony, and therefore, the locations and sizes of stones (larger than 10 cm in diameter) were also recorded.

The collected samples (litter and soil) were immediately cooled with dry ice in containers, transported to our laboratory, and then dried to a constant weight at room temperature. The litter samples were finely chopped using a mixer to obtain homogenized samples after removing coarse woody debris (fallen branches and twigs). The soil samples were sieved through a 2-mm mesh.

### Radiocesium analysis

The activity concentrations of ^137^Cs and ^134^Cs in the litter and soil (<2 mm) samples were determined using gamma ray spectrometry, and their values were expressed in activity per unit dry weight (Bq kg^−1^ dw). Samples (or subsamples) were sealed in plastic tubes (5-cm diameter, 7-cm height) and analyzed for ^137^Cs and ^134^Cs using a high-purity coaxial germanium detector (model GEM25P4–70, ORTEC, USA) at the Nuclear Science and Engineering Center of the Japan Atomic Energy Agency (JAEA). The detector was calibrated with standard gamma sources (each with a relative uncertainty of ~5% for ^137^Cs) with different sample heights. The measurement times were 2,000–20,000 s for litter samples and 2,000–100,000 s for soil samples, which allowed us to obtain both ^137^Cs and ^134^Cs concentration values with relative errors of < 10% (with some exceptions for samples having low ^134^Cs concentrations). The activity concentrations were corrected for radioactive decay to the sampling date.

Radiocesium inventories in the L and F layers, *I* (Bq m^−2^), were estimated as


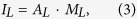



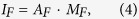


where *I* is the radiocesium inventory (Bq m^−2^) in the layer, *A* is the radiocesium activity concentration (Bq kg^−1^ dw) of the samples collected from the layer, and *M* is the amount of litter materials per unit area (kg m^−2^) in the layer. The subscripts *L* and *F* indicate the L and F layers, respectively. The radiocesium inventory in the topsoil (0–5 cm) layer was estimated to be


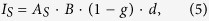


where *I*_*S*_ is the radiocesium inventory (Bq m^−2^) in the topsoil layer*, A*_*S*_ is the radiocesium activity concentration (Bq kg^−1^ dw) of the soil (<2 mm) samples, *B* is the bulk density of the soil (kg m^−3^), *g* is the gravel (>2 mm) content of the bulk soil (kg kg^−1^), and *d* is the thickness (m) of the layer (i.e., 0.05 m). Soil samples were collected and analyzed in triplicate (see above), and therefore, the radiocesium inventory in the topsoil layer at each sampling location was evaluated as the average of the triplicate samples (n = 3).

### Carbon and nitrogen analysis

A portion of each sample (both litter and soil) was analyzed for their total C and N contents using an elemental analyzer (vario PYRO cube, Elementar, Germany)^20^.

### *In situ* measurement of spatial distribution of the gamma-ray dose rate

The spatial distribution of the gamma-ray dose rate in the 20 m × 20 m square plot was measured using a PSF detection system. This system consists of a 10-m-long PSF that responds to gamma radiation, light detection units connected to both sides of the PSF, a signal processing unit, and other components^50^. The system can provide numerical values of the gamma-ray dose rate in air along the 10-m-long PSF at an interval of 0.5 m.

The 20 m × 20 m square plot established at each site was divided into two 10 m × 20 m areas. For both areas, the distributions of the gamma-ray dose rate at the ground surface in the short axis (10 m) direction were measured by successively setting the 10-m-long PSF on the forest floor at an interval of 1 m along the long axis (20 m) direction (i.e., measurements were conducted 20 × 2 times for each site). We also measured the gamma-ray dose rates at several points within the plot using a NaI scintillation survey meter. Comparing the data obtained by the PSF detection system to those obtained by the NaI survey meter allowed us to perform an *in situ* calibration of the PSF detection system.

### Visualizing the spatial distributions

To visualize the spatial distributions of the ^137^Cs inventories and the gamma-ray dose rates in the 20 m × 20 m plot areas, contour maps were created using the observation results and ordinary Kriging with the Surfer^®^ 12 software (Golden Software, Inc., Golden, CO, USA)^28^,^51^,^52^.

## Additional Information

**How to cite this article**: Koarashi, J. *et al*. Forest type effects on the retention of radiocesium in organic layers of forest ecosystems affected by the Fukushima nuclear accident. *Sci. Rep.* **6**, 38591; doi: 10.1038/srep38591 (2016).

**Publisher's note:** Springer Nature remains neutral with regard to jurisdictional claims in published maps and institutional affiliations.

## Supplementary Material

Supplementary Information

## Figures and Tables

**Table 1:** Radiocesium, carbon, and nitrogen contents in the organic and topsoil layers within the 20 m × 20 m plots in two contrasting forests.

Layer^a^	^137^Cs concentration (kBq kg^−1^ dw)	^134^Cs/^137^Cs activity ratio	^137^Cs inventory (kBq m^−2^)	% of total ^137^Cs inventory	C content (gC kg^−1^ dw)	C/N ratio
*Deciduous broad-leaved forest (DBF)*						
L layer	4.2 ± 3.0 (1.1–10.6)^c^	0.57 ± 0.03^c^,^d^	3.3 ± 2.8 (0.5–9.6)^c^	7.1 ± 6.2 (1.0–23.8)^c^	452 ± 13^c^	29.5 ± 3.0^c^
F layer	19.7 ± 3.9 (9.3–25.7)	0.56 ± 0.02	6.7 ± 2.3 (2.8–12.9)	14.1 ± 5.6 (5.5–28.4)	357 ± 48	20.1 ± 2.0
Topsoil^b^	1.5 ± 0.5 (0.9–2.8)	0.56 ± 0.02	39.8 ± 12.2 (23.6–70.4)	78.8 ± 8.4 (61.3–93.0)	54 ± 12	15.4 ± 1.1
Total	NA^e^	NA	49.8 ± 11.0 (35.0–75.7)	100	NA	NA
*Coniferous forest (CF)*						
L layer	11.6 ± 2.8 (8.0–21.1)	0.47 ± 0.01	14.6 ± 3.8 (7.0–23.9)	34.5 ± 9.7 (16.8–52.8)	440 ± 16	26.4 ± 2.3
F layer	13.2 ± 3.5 (8.3–21.2)	0.47 ± 0.01	8.4 ± 2.8 (3.9–17.3)	19.5 ± 5.6 (9.2–35.2)	362 ± 49	21.1 ± 1.5
Topsoil^b^	1.2 ± 0.6 (0.6–2.7)	0.44 ± 0.02	20.9 ± 10.6 (10.2–54.8)	46.0 ± 12.2 (22.6–70.0)	144 ± 21	16.4 ± 0.8
Total	NA	NA	43.9 ± 12.1 (27.0–78.3)	100	NA	NA

^a^Sample collections were conducted in December 2012 at the DBF site and in August 2013 at the CF site.

^b^Topsoil: the 0–5 cm layer in the mineral soil.

^c^The mean ± standard deviation of samples collected at 25 locations (n = 25) in the 20 m × 20 m plot area. The numbers in parentheses indicate the range of the values.

^d^The ^134^Cs/^137^Cs activity ratios corresponded well to the ratios theoretically predicted for Fukushima-derived radiocesium at the time of sample collection. See text.

^e^NA: Not available.

**Table 2:** Correlation coefficients between the selected variables from the 20 m × 20 m plots in the two forests.

Variables^a^	Deciduous broad-leaved forest (DBF)			Coniferous forest (CF)			
	Total ^137^Cs	Org. ^137^Cs	LMI	Total ^137^Cs	Org. ^137^Cs	LMI	
Org. ^137^Cs	−0.25			0.49*			
LMI	−0.23	0.66**		0.34	0.63**		
SBA	−0.07	0.00	0.23	−0.22	−0.17	−0.11	

Statistically significant (*P < 0.05, **P < 0.001) correlations are shown in boldface.

^a^Total ^137^Cs: Total ^137^Cs inventory (kBq m^−2^); Org. ^137^Cs: Organic-layer ^137^Cs inventory (kBq m^−2^); LMI: Litter-material inventory in the organic layer (kg m^−2^); SBA: Stand basal area (cm^2^/subplot).

## References

[b1] Hashimoto S., Ugawa S., Nanko K. & Shichi K. The total amounts of radioactively contaminated materials in forests in Fukushima, Japan. Sci. Rep. 2, 416, 10.1038/srep00416 (2012). 22639724 PMC3360326

[b2] International Atomic Energy Agency. Environmental Consequences of the Chernobyl Accident and their Remediation: Twenty Years of Experience (International Atomic Energy Agency, 2006).

[b3] Strebl F., Gerzabek M. H., Bossew P. & Kienzl K. Distribution of radiocaesium in an Austrian forest stand. Sci. Total Environ. 226, 75–83 (1999). 10077876 10.1016/s0048-9697(98)90051-1

[b4] Fesenko S. V. . ^137^Cs availability for soil to understory transfer in different types of forest ecosystems. Sci. Total Environ. 269, 87–103 (2001). 11305346 10.1016/s0048-9697(00)00818-4

[b5] Kruyts N. & Delvaux B. Soil organic horizons as a major source for radiocesium biorecycling in forest ecosystems. J. Environ. Radioact. 58, 175–190 (2002). 11814165 10.1016/s0265-931x(01)00065-0

[b6] Steiner M., Linkov I. & Yoshida S. The role of fungi in the transfer and cycling of radionuclides in forest ecosystems. J. Environ. Radioact. 58, 217–241 (2002). 11814167 10.1016/s0265-931x(01)00067-4

[b7] Konopleva I., Klemt E., Konoplev A. & Zibold G. Migration and bioavailability of ^137^Cs in forest soil of southern Germany. J. Environ. Radioact. 100, 315–321 (2009). 19167790 10.1016/j.jenvrad.2008.12.010

[b8] Sawhney B. L. Selective sorption and fixation of cations by clay minerals: a review. Clay Clay Miner. 20, 93–100 (1972).

[b9] Cremers A., Elsen A., De Preter P. & Maes A. Quantitative analysis of radiocaesium retention in soils. Nature 355, 247–249 (1988).

[b10] Valcke E. & Cremers A. Sorption-desorption dynamics of radiocaesium in organic matter soils. Sci. Total Environ. 157, 275–283 (1994).

[b11] Bunzl K., Schimmack W., Krouglov S. V. & Alexakhin R. M. Changes with time in the migration of radiocesium in the soil, as observed near Chernobyl and in Germany, 1986–1994. Sci. Total Environ. 175, 49–56 (1995).

[b12] Rosén K., Öborn I. & Lönsjö H. Migration of radiocaesium in Swedish soil profiles after the Chernobyl accident, 1987–1995. J. Environ. Radioact. 46, 45–66 (1999).

[b13] Ehlken S. & Kirchner G. Seasonal variations in soil-to-plant transfer of fallout strontium and cesium and of potassium in north German soils. J. Environ. Radioact. 33, 147–181 (1996).

[b14] Sanchez A. L. . High plant uptake of radiocesium from organic soils due to Cs mobility and low soil K content. Environ. Sci. Technol. 33, 2752–2757 (1999).

[b15] Kato H., Onda Y. & Gomi T. Interception of the Fukushima reactor accident-derived ^137^Cs, ^134^Cs and ^131^I by coniferous forest canopies. Geophys. Res. Lett. 39, L20403 (2012).

[b16] Kato H. . Temporal changes in radiocesium deposition in various forest stands following the Fukushima Dai-ichi Nuclear Power Plant accident. J. Environ. Radioact. 10.1016/j.jenvrad.2015.04.016 (2015). 26021767

[b17] Forestry Agency of Japan. Results of Surveys of Distributions of Raioactive Elements in Forest Ecosystems (in Japanese). http://www.rinya.maff.go.jp/j/press/kenho/130329.html (2013).

[b18] Nakanishi T., Matsunaga T., Koarashi J. & Atarashi-Andoh M. ^137^Cs vertical migration in a deciduous forest soil following the Fukushima Dai-ichi Nuclear Power Plant accident. J. Environ. Radioact. 128, 9–14 (2014). 24239654 10.1016/j.jenvrad.2013.10.019

[b19] Koarashi J. . Quantitative aspects of heterogeneity in soil organic matter dynamics in a cool-temperate Japanese beech forest: a radiocarbon-based approach. Glob. Change Biol. 15, 631–642 (2009).

[b20] Koarashi J., Atarashi-Andoh M., Takeuchi E. & Nishimura S. Topographic heterogeneity effect on the accumulation of Fukushima-derived radiocesium on forest floor driven by biologically mediated processes. Sci. Rep. 4, 6853, 10.1038/srep06853 (2014). 25358420 PMC4215300

[b21] Orwa C. . Agroforestree Database: a tree reference and selection guide version 4.0. World Agroforestry Centre, Kenya. http://www.worldagroforestry.org/output/agroforestree-database (2009).

[b22] Prescott C. E., Zabek L. M., Staley C. L. & Kabzems R. Decomposition of broadleaf and needle litter in forests of British Columbia: influences of litter type, forest type, and litter mixtures. Can. J. For. Res. 30, 1742–1750 (2000).

[b23] Zhang D., Hui D., Luo Y. & Zhou G. Rates of litter decomposition in terrestrial ecosystems: global patterns and controlling factors. J. Plant Ecol. 1, 85–93 (2008).

[b24] Hashimoto S. . Predicted spatio-temporal dynamics of radiocesium deposited onto forests following the Fukushima nuclear accident. Sci. Rep. 3, 2564, 10.1038/srep02564 (2013). 23995073 PMC3759142

[b25] Koarashi J. . Post-deposition early-phase migration and retention behavior of radiocesium in a litter–mineral soil system in a Japanese deciduous forest affected by the Fukushima nuclear accident. Chemosphere 165, 335–341 (2016). 27664523 10.1016/j.chemosphere.2016.09.043

[b26] Ota M., Nagai H. & Koarashi J. Modeling dynamics of ^137^Cs in forest surface environments: Application to a contaminated forest site near Fukushima and assessment of potential impacts of soil organic matter interactions. Sci. Total Environ. 551–552, 590–604 (2016). 10.1016/j.scitotenv.2016.02.068 26897402

[b27] Uriarte M., Turner B. L., Thompson J. & Zimmerman J. K. Linking spatial patterns of leaf litterfall and soil nutrients in a tropical forest: a neighborhood approach. Ecol. Appl. 25, 2022–2034 (2015). 26591466 10.1890/15-0112.1

[b28] Karadeniz Ö. . Persistence of ^137^Cs in the litter layers of forest soil horizoons of Mount IDA/Kazdagi, Turkey. J. Environ. Radioact. 139, 125–134 (2015). 25464048 10.1016/j.jenvrad.2014.10.004

[b29] Koarashi J. . Factors affecting vertical distribution of Fukushima accident-derived radiocesium in soil under different land-use conditions. Sci. Total Environ. 431, 392–401 (2012). 22706146 10.1016/j.scitotenv.2012.05.041

[b30] Matsunaga T. . Comparison of the vertical distributions of Fukushima nuclear accident radiocesium in soil before and after the first rainy season, with physicochemical and mineralogical interpretations. Sci. Total Environ. 447, 301–314 (2013). 23391896 10.1016/j.scitotenv.2012.12.087

[b31] Ono K. . Organic carbon accumulation processes on a forest floor during an early humification stage in a temperate deciduous forest in Japan: Evaluations of chemical compositional changes by ^13^C NMR and their decomposition rates from litterbag experiment. Geoderma 151, 351–356 (2009).

[b32] Bonanomi G. . Decomposition and nutrient dynamics in mixed litter of Mediterranean species. Plant Soil 331, 481–496 (2010).

[b33] Attiwill P. M. & Adams M. A. Tansley Review No. 50. Nutrient cycling in forests. New Phytol. 124, 561–582 (1993). 10.1111/j.1469-8137.1993.tb03847.x 33874438

[b34] Shcheglov A. I. Dynamics of radionuclide redistribution and pathways in forest environments: long-term field research in different landscapes In Contaminated Forests (eds. Linkov I. L. & Schell W. R.) 23–39 (Kluwer Academic Publishers, 1999).

[b35] Fujii K. . Vertical migration of radiocesium and clay mineral composition in five forest soils contaminated by the Fukushima nuclear accident. Soil Sci. Plant Nutr. 60, 751–764 (2014).

[b36] Thiry Y. . Impact of Scots pine (*Pinus sylvestri*s L.) plantings on long term ^137^Cs and ^90^Sr recycling from a waste burial site in the Chernobyl Red Forest. J. Environ. Radioact. 100, 1062–1068 (2009). 19525043 10.1016/j.jenvrad.2009.05.003

[b37] Tagami K., Uchida S., Ishii N. & Kagiya S. Translocation of radiocesium from stems and leaves of plants and the effect on radiocesium concentrations in newly emerged plant tissues. J. Environ. Radioact. 111, 65–69 (2012). 22027214 10.1016/j.jenvrad.2011.09.017

[b38] Yoshihara T. . Changes in radiocesium contamination from Fukushima in foliar parts of 10 common tree species in Japan between 2011 and 2013. J. Environ. Radioact. 138, 220–226 (2014). 25261868 10.1016/j.jenvrad.2014.09.002

[b39] Kajimoto T. . Dynamics of radiocesium in forest ecosystems affected by the Fukushima Daiichi nuclear power plant accident: Species-related transfer processes of radiocesium from tree crowns to ground floor during the first two years. J. Jpn. For. Soc. 97, 33–43 (2015) (in Japanese with English abstract).

[b40] Rafferty B., Brennan M., Dawson D. & Dowding D. Mechanisms of ^137^Cs migration in coniferous forest soils. J. Environ. Radioact. 48, 131–143 (2000).

[b41] Atarashi-Andoh M., Koarashi J., Ishizuka S. & Hirai K. Seasonal patterns and control factors of CO_2_ effluxes from surface litter, soil organic carbon, and toot-derived carbon estimated using radiocarbon signatures. Agric. For. Meteorol. 152, 149–158 (2012).

[b42] Fröberg M. . Mean residence time of O horizon carbon along a climate gradient in Scandinavia estimated by ^14^C measurements of archived soils. Biogeochem. 104, 227–236 (2011).

[b43] Fisher S. G. & Likens G. E. Energy flow in Bear Brook, New Hampshire: An integrative approach to stream ecosystem metabolism. Ecol. Monogr. 43, 421–439 (1973).

[b44] Ferrari J. B. & Sugita S. A spatially explicit model of leaf litter fall in hemlock-hardwood forests. Can. J. For. Res. 26, 1905–1913 (1996).

[b45] Dwyer L. M. & Merriam G. Influence of topographic heterogeneity on deciduous litter decomposition. Oikos 37, 228–237 (1981).

[b46] Zhang W. . Earthworms facilitate carbon sequestration through unequal amplification of carbon stabilization compared with mineralization. Nat. Commun. 4, 2576, 10.1038/ncomms3576 (2013). 24129390

[b47] Miller K. M., Kuiper J. L. & Helfer I. K. ^137^Cs fallout depth distributions in forest versus field sites: Implications for external gamma dose rates. J. Environ. Radioact. 12, 23–47 (1990).

[b48] National Land Agency, Japan. Tochi-Bunrui-Zu Fukushima-ken (Land Classification Map for Fukushima Prefecture) (Japan Map Center, 1972).

[b49] Koarashi J. . Retention of potentially mobile radiocesium in forest surface soils affected by the Fukushima nuclear accident. Sci. Rep. 2, 1005, 10.1038/srep01005 (2012). 23256039 PMC3525936

[b50] Gamo H. . Development of a PSF-detector for contaminated areas. Prog. Nucl. Sci. Technol. 4, 695–698 (2014).

[b51] Mabit L. & Bernard C. Assessment of spatial distribution of fallout radionuclides through geostatistics concept. J. Environ. Radioact. 97, 206–219 (2007). 17673340 10.1016/j.jenvrad.2007.05.008

[b52] Atarashi-Andoh M. . Catchment-scale distribution of radiocesium air dose rate in a mountainous deciduous forest and its relation to topography. J. Environ. Radioact. 147, 1–7 (2015). 26005183 10.1016/j.jenvrad.2015.05.004

[b53] Ministry of Education, Culture, Sports, Scienceγ, and Technology, Japan. Extension Site of Distribution Map of Radiation Dose, etc.,/GSI Maps. http://ramap.jmc.or.jp/map/eng/ (2011).

