# Supplementary material for: Forest type effects on the retention of radiocesium in organic layers of forest ecosystems affected by the Fukushima nuclear accident
Source: Sci Rep. 2016 Dec 15;6:38591. doi: 10.1038/srep38591 (PMC5156898; doi:10.1038/srep38591)
Supplement: Supplementary Information [file srep38591-s1.pdf]

Supplementary information

Title: Forest type effects on the retention of radiocesium in organic layers of forest ecosystems affected by the Fukushima nuclear accident

Authors: Koarashi, J., Atarashi-Andoh M., Matsunaga, T. & Sanada, Y.

Table S1. Radiocesium in organic (L and F) and topsoil (0–5 cm) layers within a 20 m × 20 m plot in the DBF.

Location	Layer	Litter density[a] (kg m^{-2})	Bulk density[b] (g cm^{-3})	Gravel content[b] (%)	Radiocesium concentration[c] ^{137}Cs (kBq kg^{-1} dw)	^{134}Cs (kBq kg^{-1} dw)	^{134}Cs/^{137}Cs	^{137}Cs inventory (kBq m^{-2})[c]	% of total inventory
DBF-1	L	0.89	NA[d]	NA	4.52 ± 0.13	2.69 ± 0.13	0.59	4.0 ± 0.1	10.2
	F	0.55	NA	NA	18.39 ± 0.31	10.37 ± 0.32	0.56	10.1 ± 0.2	25.7
	Soil	NA	0.52 (0.13)	9.7 (5.6)	0.91 (0.74)	0.48 (0.41)	0.53	25.2 (26.4)	64.1
DBF-2	L	0.50	NA	NA	1.07 ± 0.05	0.57 ± 0.05	0.53	0.5 ± 0.0	1.0
	F	0.32	NA	NA	24.83 ± 0.43	14.02 ± 0.42	0.56	8.0 ± 0.1	15.6
	Soil	NA	0.47 (0.04)	10.5 (7.4)	2.09 (1.00)	1.20 (0.67)	0.58	42.6 (13.1)	83.3
DBF-3	L	0.78	NA	NA	2.65 ± 0.12	1.49 ± 0.12	0.56	2.1 ± 0.1	5.9
	F	0.21	NA	NA	19.80 ± 0.44	10.63 ± 0.43	0.54	4.2 ± 0.1	12.1
	Soil	NA	0.53 (0.07)	3.7 (2.8)	1.15 (0.52)	0.61 (0.32)	0.53	28.7 (9.7)	82.0
DBF-4	L	0.85	NA	NA	9.08 ± 0.19	5.30 ± 0.20	0.58	7.7 ± 0.2	11.4
	F	0.28	NA	NA	20.44 ± 0.43	11.15 ± 0.42	0.55	5.7 ± 0.1	8.4
	Soil	NA	0.54 (0.12)	3.4 (0.3)	2.27 (1.75)	1.31 (1.07)	0.58	54.1 (31.9)	80.2
DBF-5	L	0.67	NA	NA	1.86 ± 0.10	1.00 ± 0.10	0.54	1.2 ± 0.1	2.3
	F	0.57	NA	NA	18.25 ± 0.26	10.29 ± 0.26	0.56	10.4 ± 0.1	19.5
	Soil	NA	0.61 (0.05)	2.0 (0.7)	1.32 (1.22)	0.72 (0.66)	0.54	41.8 (41.9)	78.1

DBF-6	L	0.71	NA	NA	1.90 ± 0.08	1.03 ± 0.08	0.54	1.3 ± 0.1	3.0
	F	0.65	NA	NA	19.79 ± 0.27	11.47 ± 0.27	0.58	12.9 ± 0.2	28.4
	Soil	NA	0.49 (0.03)	8.0 (5.3)	1.37 (0.64)	0.75 (0.34)	0.55	31.3 (13.9)	68.6
DBF-7	L	0.64	NA	NA	3.45 ± 0.08	1.93 ± 0.08	0.56	2.2 ± 0.1	3.9
	F	0.27	NA	NA	25.66 ± 0.47	14.67 ± 0.48	0.57	6.8 ± 0.1	11.9
	Soil	NA	0.51 (0.11)[e]	5.4 (0.4)[e]	2.07 (0.94)[e]	1.15 (0.47)[e]	0.56	48.3 (11.9)[e]	84.2
DBF-8	L	0.90	NA	NA	6.04 ± 0.17	3.74 ± 0.18	0.62	5.4 ± 0.2	11.4
	F	0.26	NA	NA	20.57 ± 0.44	12.12 ± 0.45	0.59	5.4 ± 0.1	11.5
	Soil	NA	0.56 (0.12)	2.3 (0.5)	1.48 (1.20)	0.82 (0.70)	0.56	36.5 (25.1)	77.1
DBF-9	L	0.84	NA	NA	7.08 ± 0.18	3.91 ± 0.17	0.55	5.9 ± 0.1	11.9
	F	0.23	NA	NA	20.20 ± 0.37	10.97 ± 0.36	0.54	4.7 ± 0.1	9.5
	Soil	NA	0.53 (0.04)	2.9 (0.8)	1.47 (1.20)	0.79 (0.66)	0.53	39.2 (33.5)	78.6
DBF-10	L	0.86	NA	NA	10.60 ± 0.19	6.02 ± 0.19	0.57	9.2 ± 0.2	23.6
	F	0.28	NA	NA	20.62 ± 0.39	10.90 ± 0.38	0.53	5.7 ± 0.1	14.9
	Soil	NA	0.47 (0.15)	2.2 (1.0)	1.09 (0.37)	0.63 (0.23)	0.58	23.6 (2.9)	61.3
DBF-11	L	0.72	NA	NA	4.78 ± 0.15	2.76 ± 0.15	0.58	3.5 ± 0.1	7.7
	F	0.49	NA	NA	15.97 ± 0.27	8.67 ± 0.26	0.54	7.8 ± 0.1	17.4
	Soil	NA	0.77 (0.08)	1.4 (0.3)	0.87 (0.08)	0.45 (0.10)	0.52	33.6 (6.7)	74.8
DBF-12	L	0.51	NA	NA	1.33 ± 0.10	0.84 ± 0.10	0.63	0.7 ± 0.0	1.0
	F	0.28	NA	NA	15.79 ± 0.33	9.08 ± 0.33	0.57	4.4 ± 0.1	6.8
	Soil	NA	0.58 (0.08)	2.5 (1.3)	2.14 (0.37)	1.22 (0.18)	0.57	60.5 (6.6)	92.2
DBF-13	L	0.68	NA	NA	1.63 ± 0.06	0.94 ± 0.06	0.58	1.1 ± 0.0	1.5
	F	0.22	NA	NA	19.03 ± 0.53	9.92 ± 0.52	0.52	4.2 ± 0.1	5.5

	Soil	NA	0.61 (0.07)	3.9 (0.6)	2.52 (2.13)	1.45 (1.25)	0.58	70.4 (50.1)	93.0
DBF-14	L	0.67	NA	NA	8.48 ± 0.24	4.78 ± 0.24	0.56	5.6 ± 0.2	14.0
	F	0.25	NA	NA	23.40 ± 0.56	13.95 ± 0.57	0.60	5.9 ± 0.1	14.7
	Soil	NA	0.55 (0.06)	1.8 (0.4)	1.02 (0.75)	0.54 (0.43)	0.53	28.6 (22.4)	71.3
DBF-15	L	1.02	NA	NA	5.32 ± 0.14	3.22 ± 0.14	0.60	5.4 ± 0.1	9.4
	F	0.25	NA	NA	23.41 ± 0.52	14.06 ± 0.54	0.60	5.8 ± 0.1	10.0
	Soil	NA	0.53 (0.09)	2.4 (0.8)	1.73 (0.28)	1.02 (0.16)	0.59	46.3 (14.9)	80.6
DBF-16	L	0.40	NA	NA	1.18 ± 0.03	0.64 ± 0.03	0.54	0.5 ± 0.0	1.2
	F	0.39	NA	NA	22.12 ± 0.48	11.98 ± 0.47	0.54	8.6 ± 0.2	21.2
	Soil	NA	0.49[f]	2.2[f]	1.28 ± 0.05[f]	0.67 ± 0.05[f]	0.52	31.4 ± 1.2[f]	77.6
DBF-17	L	0.69	NA	NA	5.30 ± 0.18	2.85 ± 0.17	0.54	3.6 ± 0.1	10.2
	F	0.23	NA	NA	22.39 ± 0.49	12.52 ± 0.51	0.56	5.2 ± 0.1	14.5
	Soil	NA	0.63 (0.10)	2.9 (1.0)	0.87 (0.02)	0.49 (0.02)	0.56	27.1 (3.6)	75.4
DBF-18	L	0.58	NA	NA	1.51 ± 0.12	0.92 ± 0.13	0.61	0.9 ± 0.1	1.2
	F	0.31	NA	NA	22.51 ± 0.46	12.14 ± 0.45	0.54	7.0 ± 0.1	9.5
	Soil	NA	0.47 (0.09)	4.1 (2.3)	2.78 (2.11)	1.54 (1.17)	0.56	65.2 (57.9)	89.3
DBF-19	L	0.48	NA	NA	1.75 ± 0.12	1.03 ± 0.12	0.59	0.8 ± 0.1	1.6
	F	0.40	NA	NA	23.81 ± 0.51	13.48 ± 0.51	0.57	9.5 ± 0.2	18.1
	Soil	NA	0.62 (0.13)	1.2 (0.4)	1.51 (1.20)	0.87 (0.67)	0.58	42.3 (31.0)	80.4
DBF-20	L	0.68	NA	NA	3.96 ± 0.17	2.44 ± 0.18	0.61	2.7 ± 0.1	6.1
	F	0.34	NA	NA	18.83 ± 0.40	10.76 ± 0.40	0.57	6.5 ± 0.1	14.6
	Soil	NA	0.66 (0.09)	1.5 (0.2)	1.05 (0.24)	0.61 (0.17)	0.58	35.1 (9.9)	79.3
DBF-21	L	0.54	NA	NA	2.73 ± 0.13	1.45 ± 0.13	0.53	1.5 ± 0.1	3.0

	F	0.52	NA	NA	15.34 ± 0.33	8.82 ± 0.33	0.58	8.0 ± 0.2	16.3
	Soil	NA	0.82 (0.10)	2.8 (2.2)	0.98 (0.14)	0.54 (0.11)	0.55	39.4 (2.6)	80.7
DBF-22	L	1.02	NA	NA	4.93 ± 0.15	2.68 ± 0.15	0.54	5.0 ± 0.2	10.7
	F	0.47	NA	NA	13.17 ± 0.27	7.93 ± 0.27	0.60	6.2 ± 0.1	13.1
	Soil	NA	0.55 (0.14)	3.4 (1.4)	1.43 (0.79)	0.82 (0.47)	0.58	35.9 (12.5)	76.2
DBF-23	L	0.58	NA	NA	1.08 ± 0.09	0.55 ± 0.10	0.51	0.6 ± 0.1	1.3
	F	0.30	NA	NA	9.26 ± 0.29	5.41 ± 0.29	0.58	2.8 ± 0.1	6.0
	Soil	NA	0.66 (0.15)	0.7 (0.3)	1.40 (0.86)	0.79 (0.58)	0.57	43.1 (18.0)	92.7
DBF-24	L	0.91	NA	NA	10.52 ± 0.23	5.87 ± 0.23	0.56	9.6 ± 0.2	20.7
	F	0.30	NA	NA	22.72 ± 0.47	12.27 ± 0.47	0.54	6.8 ± 0.1	14.7
	Soil	NA	0.66 (0.11)	1.5 (0.2)	0.98 (0.75)	0.57 (0.42)	0.57	29.9 (20.0)	64.6
DBF-25	L	0.80	NA	NA	2.11 ± 0.13	1.15 ± 0.13	0.55	1.7 ± 0.1	4.0
	F	0.33	NA	NA	16.05 ± 0.38	8.87 ± 0.38	0.55	5.2 ± 0.1	12.4
	Soil	NA	0.60 (0.09)	2.6 (0.8)	1.24 (0.74)	0.64 (0.42)	0.52	35.3 (18.3)	83.6

[a]Not including coarse woody debris (fallen branches and twigs).

[b]The mean and standard deviation are shown in parenthesis for three replicate samples (n = 3).

[c]For the organic (L and F) layers, errors represent the counting errors in the radiation measurement. For the soil layers, the mean and standard deviation are shown in parenthesis for three replicate samples (n = 3).

[d]NA: Not available.

[e]The mean and standard deviation are shown in parenthesis for two replicate samples (n = 2) due to the stony topsoil at this location (DBF-7).

[f]There was no replicate sample (n = 1) due to the stony topsoil at this location (DBF-16).

Table S2. Radiocesium in organic (L and F) and topsoil (0–5 cm) layers within a 20 m × 20 m plot in the CF.

Location	Layer	Litter density[a] (kg m^{-2})	Bulk density[b] (g cm^{-3})	Gravel content[b] (%)	Radiocesium concentration[c] ^{137}Cs (kBq kg^{-1} dw)	^{134}Cs (kBq kg^{-1} dw)	$^{134}Cs/^{137}Cs$	^{137}Cs inventory (kBq m^{-2})[c]	% of total inventory
CF-1	L	1.72	NA[d]	NA	13.86 ± 0.16	6.48 ± 0.13	0.47	23.9 ± 0.3	52.8
	F	0.76	NA	NA	14.75 ± 0.15	6.81 ± 0.12	0.46	11.1 ± 0.1	24.7
	Soil	NA	0.36 (0.02)	3.5 (1.9)	0.58 (0.44)	0.26 (0.23)	0.44	10.2 (7.5)	22.6
CF-2	L	1.61	NA	NA	9.64 ± 0.14	4.49 ± 0.11	0.47	15.5 ± 0.2	48.4
	F	0.52	NA	NA	9.56 ± 0.11	4.71 ± 0.09	0.49	5.0 ± 0.1	15.6
	Soil	NA	0.31 (0.04)	4.2 (2.0)	0.76 (0.34)	0.33 (0.16)	0.43	11.5 (5.2)	36.0
CF-3	L	1.13	NA	NA	9.43 ± 0.12	4.46 ± 0.09	0.47	10.7 ± 0.1	25.5
	F	0.46	NA	NA	8.34 ± 0.09	3.80 ± 0.07	0.46	3.9 ± 0.0	9.2
	Soil	NA	0.34 (0.03)	2.5 (0.6)	1.55 (1.68)	0.72 (0.81)	0.46	27.3 (30.8)	65.2
CF-4	L	1.25	NA	NA	12.08 ± 0.14	5.53 ± 0.11	0.46	15.2 ± 0.2	27.9
	F	0.68	NA	NA	16.15 ± 0.15	7.53 ± 0.12	0.47	10.9 ± 0.1	20.2
	Soil	NA	0.27 (0.02)	10.1 (2.2)	2.17 (1.75)	1.00 (0.79)	0.46	28.2 (24.9)	51.9
CF-5	L	0.94	NA	NA	17.38 ± 0.17	8.17 ± 0.13	0.47	16.3 ± 0.2	33.3
	F	0.81	NA	NA	21.23 ± 0.18	9.83 ± 0.14	0.46	17.3 ± 0.1	35.2
	Soil	NA	0.33 (0.05)	1.6 (0.6)	1.03 (0.82)	0.44 (0.38)	0.43	15.4 (9.5)	31.5
CF-6	L	0.78	NA	NA	8.97 ± 0.12	4.10 ± 0.10	0.46	7.0 ± 0.1	24.9
	F	0.69	NA	NA	8.56 ± 0.10	3.95 ± 0.08	0.46	5.9 ± 0.1	21.0
	Soil	NA	0.37 (0.07)	2.0 (0.6)	0.83 (0.14)	0.37 (0.06)	0.45	15.2 (3.8)	54.1
CF-7	L	1.08	NA	NA	13.15 ± 0.14	6.24 ± 0.11	0.47	14.2 ± 0.2	38.9

	F	0.76	NA	NA	12.03 ± 0.13	5.49 ± 0.10	0.46	9.2 ± 0.1	25.1
	Soil	NA	0.35 (0.08)	2.8 (1.1)	0.71 (0.38)	0.31 (0.20)	0.44	13.1 (9.7)	36.0
CF-8	L	1.55	NA	NA	10.31 ± 0.14	4.66 ± 0.11	0.45	16.0 ± 0.2	26.2
	F	0.50	NA	NA	17.38 ± 0.16	8.33 ± 0.13	0.48	8.7 ± 0.1	14.3
	Soil	NA	0.30 (0.02)	10.5 (4.3)	2.67 (1.84)	1.23 (0.88)	0.46	36.2 (26.6)	59.5
CF-9	L	1.41	NA	NA	11.82 ± 0.16	5.60 ± 0.13	0.47	16.7 ± 0.2	42.5
	F	0.54	NA	NA	12.59 ± 0.15	5.91 ± 0.12	0.47	6.9 ± 0.1	17.5
	Soil	NA	0.29 (0.03)	2.3 (1.5)	1.10 (0.87)	0.51 (0.44)	0.47	15.7 (12.0)	40.0
CF-10	L	1.10	NA	NA	7.96 ± 0.12	3.88 ± 0.10	0.49	8.7 ± 0.1	20.9
	F	0.58	NA	NA	15.70 ± 0.16	7.40 ± 0.13	0.47	9.1 ± 0.1	21.9
	Soil	NA	0.30 (0.04)	2.5 (0.3)	1.65 (0.65)	0.73 (0.29)	0.44	23.9 (8.3)	57.2
CF-11	L	1.00	NA	NA	21.08 ± 0.20	9.79 ± 0.16	0.46	21.0 ± 0.2	52.0
	F	0.57	NA	NA	13.28 ± 0.15	6.16 ± 0.12	0.46	7.6 ± 0.1	18.7
	Soil	NA	0.35 (0.06)	1.7 (1.3)	0.69 (0.08)	0.28 (0.04)	0.40	11.8 (1.5)	29.3
CF-12	L	1.10	NA	NA	12.60 ± 0.15	5.94 ± 0.12	0.47	13.9 ± 0.2	30.1
	F	0.66	NA	NA	8.33 ± 0.11	3.76 ± 0.08	0.45	5.5 ± 0.1	12.0
	Soil	NA	0.35 (0.07)	1.7 (1.1)	1.55 (0.81)	0.68 (0.37)	0.44	26.7 (13.6)	57.9
CF-13	L	1.24	NA	NA	12.33 ± 0.14	5.61 ± 0.11	0.45	15.2 ± 0.2	37.6
	F	0.76	NA	NA	11.27 ± 0.13	5.15 ± 0.11	0.46	8.6 ± 0.1	21.2
	Soil	NA	0.39 (0.02)	2.1 (1.2)	0.90 (1.16)	0.37 (0.52)	0.41	16.7 (21.0)	41.2
CF-14	L	0.90	NA	NA	12.77 ± 0.16	6.01 ± 0.13	0.47	11.5 ± 0.1	29.9
	F	0.62	NA	NA	11.89 ± 0.14	5.87 ± 0.12	0.49	7.4 ± 0.1	19.1
	Soil	NA	0.31 (0.05)	3.7 (2.5)	1.40 (1.13)	0.61 (0.51)	0.44	19.7 (13.8)	51.0

CF-15	L	1.11	NA	NA	11.79 ± 0.15	5.51 ± 0.12	0.47	13.1 ± 0.2	30.1
	F	0.62	NA	NA	14.06 ± 0.14	6.45 ± 0.11	0.46	8.7 ± 0.1	20.0
	Soil	NA	0.44 (0.07)	2.8 (0.5)	1.00 (0.12)	0.41 (0.03)	0.41	21.8 (2.8)	49.9
CF-16	L	1.51	NA	NA	8.74 ± 0.14	4.01 ± 0.11	0.46	13.2 ± 0.2	42.2
	F	0.63	NA	NA	9.99 ± 0.12	4.61 ± 0.10	0.46	6.3 ± 0.1	20.0
	Soil	NA	0.40 (0.04)	1.8 (0.2)	0.62 (0.45)	0.26 (0.22)	0.42	11.9 (7.5)	37.8
CF-17	L	1.74	NA	NA	10.86 ± 0.15	5.02 ± 0.12	0.46	18.9 ± 0.3	33.4
	F	0.64	NA	NA	15.24 ± 0.16	7.12 ± 0.13	0.47	9.7 ± 0.1	17.1
	Soil	NA	0.39 (0.05)	3.0 (0.6)	1.53 (1.35)	0.67 (0.60)	0.44	27.9 (21.3)	49.4
CF-18	L	1.42	NA	NA	9.52 ± 0.14	4.43 ± 0.12	0.47	13.5 ± 0.2	36.8
	F	1.02	NA	NA	10.77 ± 0.15	5.36 ± 0.12	0.50	11.0 ± 0.1	29.8
	Soil	NA	0.35 (0.06)	4.0 (1.5)	0.74 (0.18)	0.31 (0.07)	0.42	12.3 (1.8)	33.4
CF-19	L	0.86	NA	NA	10.84 ± 0.15	5.09 ± 0.12	0.47	9.3 ± 0.1	34.4
	F	0.61	NA	NA	11.16 ± 0.13	5.23 ± 0.11	0.47	6.8 ± 0.1	25.1
	Soil	NA	0.36 (0.05)	3.2 (1.2)	0.63 (0.28)	0.27 (0.15)	0.43	10.9 (4.5)	40.5
CF-20	L	1.30	NA	NA	10.12 ± 0.14	4.78 ± 0.12	0.45	13.1 ± 0.2	16.8
	F	0.52	NA	NA	20.11 ± 0.17	8.93 ± 0.14	0.44	10.4 ± 0.1	13.2
	Soil	NA	0.45 (0.02)	2.8 (0.3)	2.47 (0.87)	1.12 (0.39)	0.45	54.8 (18.9)	70.0
CF-21	L	1.97	NA	NA	9.97 ± 0.14	4.47 ± 0.12	0.45	19.6 ± 0.3	29.9
	F	1.03	NA	NA	11.98 ± 0.13	5.71 ± 0.11	0.48	12.3 ± 0.1	18.7
	Soil	NA	0.42 (0.03)	3.4 (1.2)	1.64 (0.57)	0.73 (0.29)	0.45	33.8 (13.2)	51.4
CF-22	L	1.60	NA	NA	10.82 ± 0.15	4.97 ± 0.12	0.46	17.4 ± 0.2	48.2
	F	0.42	NA	NA	15.15 ± 0.17	7.03 ± 0.14	0.46	6.4 ± 0.1	17.7

	Soil	NA	0.32 (0.03)	1.9 (0.7)	0.78 (0.18)	0.30 (0.09)	0.39	12.3 (2.5)	34.1
CF-23	L	1.40	NA	NA	10.53 ± 0.15	4.87 ± 0.12	0.46	14.7 ± 0.2	44.1
	F	0.61	NA	NA	10.29 ± 0.14	4.74 ± 0.11	0.46	6.3 ± 0.1	18.8
	Soil	NA	0.32 (0.04)	3.0 (1.3)	0.82 (0.34)	0.35 (0.14)	0.43	12.4 (4.2)	37.1
CF-24	L	1.09	NA	NA	12.33 ± 0.15	5.77 ± 0.12	0.47	13.5 ± 0.2	31.5
	F	0.44	NA	NA	14.28 ± 0.16	6.60 ± 0.13	0.46	6.3 ± 0.1	14.7
	Soil	NA	0.33 (0.05)	4.9 (3.0)	1.50 (0.64)	0.68 (0.29)	0.45	23.0 (9.3)	53.8
CF-25	L	1.25	NA	NA	10.02 ± 0.14	4.69 ± 0.11	0.47	12.6 ± 0.2	24.6
	F	0.51	NA	NA	16.72 ± 0.16	7.68 ± 0.13	0.46	8.5 ± 0.1	16.7
	Soil	NA	0.47 (0.04)	1.8 (0.4)	1.31 (0.67)	0.60 (0.32)	0.46	29.9 (13.1)	58.7

[a]Not including coarse woody debris (fallen branches and twigs).

[b]The mean and standard deviation are shown in parenthesis for three replicate samples (n = 3).

[c]For the organic (L and F) layers, errors represent the counting errors in the radiation measurement. For the soil layers, the mean and standard deviation are shown in parenthesis for three replicate samples (n = 3).

[d]NA: Not available.
